# Efficient p-type dye-sensitized solar cells with all-nano-electrodes: NiCo_2_S_4_ mesoporous nanosheet counter electrodes directly converted from NiCo_2_O_4_ photocathodes

**DOI:** 10.1186/1556-276X-9-608

**Published:** 2014-11-11

**Authors:** Zhiwei Shi, Hao Lu, Qiong Liu, Fengren Cao, Jun Guo, Kaimo Deng, Liang Li

**Affiliations:** 1 College of Physics, Optoelectronics and Energy & Collaborative Innovation Center of Suzhou Nano Science and Technology, Soochow University, Suzhou 215006, People's Republic of China; 2 Analysis and Testing Center, Soochow University, Suzhou 215006, People's Republic of China

**Keywords:** Dye-sensitized solar cells, p-type, Counter electrodes, Nanosheets, Ternary sulfides

## Abstract

We report the successful growth of NiCo_2_S_4_ nanosheet films converted from NiCo_2_O_4_ nanosheet films on fluorine-doped tin oxide substrates by a low-temperature solution process. Low-cost NiCo_2_S_4_ and NiCo_2_O_4_ nanosheet films were directly used for replacing conventional Pt and NiO as counter electrodes and photocathodes, respectively, to construct all-nano p-type dye-sensitized solar cells (p-DSSCs) with high performance. Compared to Pt, NiCo_2_S_4_ showed higher catalytic activity towards the I^-^/I_3_^-^ redox in electrolyte, resulting in an improved photocurrent density up to 2.989 mA/cm^2^, which is the highest value in reported p-DSSCs. Present p-DSSCs demonstrated a cell efficiency of 0.248 % that is also comparable with typical NiO-based p-DSSCs.

## Background

Dye-sensitized solar cells (DSSCs) are typically composed of dye-sensitized nanocrystalline semiconductors, an iodide/triiodide (I^-^/I_3_^-^) redox electrolyte, and a counter electrode (CE) [1-3]. One research direction to improve conversion efficiency of DSSCs is to construct a tandem DSSC in a sandwich configuration, which combines a photoanode from an n-type DSSC (n-DSSC) and a photocathode from a p-type DSSC (p-DSSC) [4,5]. In a tandem cell, the overall photovoltage is the sum of two parts, while the photocurrent is limited by the photoelectrode with a smaller current. Recently, the conversion efficiency of n-DSSCs reaches as high as 12% [6], but the p-DSSCs still suffers from low efficiencies and thus limit the overall efficiency of the tandem DSSCs. Although much attention has been paid to search ideal p-type semiconductor materials and efficient dyes [7-10], the best p-DSSC exhibits efficiency of only 1.3% by utilizing [Co(en)_3_]^2+/3+^ redox couple and PMI-6 T-TPA dye [11]. It is worthy to note that few results are devoted to the study of CEs for p-DSSCs, ignoring a feasible approach to further improve the conversion efficiency. The function of CEs is to transfer the electrons (holes) arriving from the external circuit to the redox electrolyte to catalyze the reduction (oxidization) of the redox couple. Generally, platinum (Pt) is the preferred CE material due to its superior electrocatalytic activity, high electrical conductivity, and excellent chemical stability. However, as a noble metal, Pt is one of the most expensive materials and has low abundance in the earth, preventing it from being used for large-scale manufacture of DSSCs.

Since Grätzel's group found that cobalt sulfide (CoS) has excellent catalytic activity for the iodine-based redox couples [12], great efforts have been made to exploit abundant low-cost substitutes for Pt, including metal sulfides, nitrides, and carbides [13-16] Unfortunately, to date, these reported catalysts can hardly compete with Pt in the performance of DSSCs. As an important class of chalcogenides, semiconducting sulfides have drawn intensive attention due to their distinctive electronic properties, interesting chemical behaviors, and a variety of applications. Particularly, binary metal sulfides (NiS and CoS) have exhibited almost the same conversion efficiency as Pt in DSSCs [17-19]. Compared with binary NiS and CoS, ternary sulfide NiCo_2_S_4_ is expected to offer richer redox reactions due to the contributions from both nickel and cobalt ions. For example, NiCo_2_S_4_ has demonstrated enhanced catalytic activity for oxygen evolution and polysulfide redox couple [20,21]. Recent results also showed that NiCo_2_S_4_ can be used as CEs of n-type DSSCs, but its efficiencies are lower than those of the Pt-based cells [22,23].

In this paper, we reported low-cost NiCo_2_S_4_ nanosheet (NS) films grown on a fluorine-doped tin oxide (FTO) substrate as a high-performance CE for p-DSSCs composed of p-type NiCo_2_O_4_ semiconductor photocathode. The NiCo_2_S_4_ NS film was synthesized via a facile two-step process including the synthesis of NiCo_2_O_4_ NS film on a FTO substrate and then an anion ion exchange process under hydrothermal reaction. When applying the NiCo_2_S_4_ as a CE and NiCo_2_O_4_ as a photocathode, novel all-nano-p-DSSCs achieved an impressive photocurrent of 2.989 mA/cm^2^ and cell efficiency of 0.248% versus 1.824 mA/cm^2^ and 0.158% for Pt under the same conditions. To the best of our knowledge, this efficiency is comparable and even higher than that of NiO-based p-DSSCs with an I^-^/I^3-^ redox couple.

## Methods

### Synthesis of NiCo_2_S_4_ nanosheet films

A two-step hydrothermal process was used to synthesize NiCo_2_S_4_ nanosheet films on FTO substrates. In the first step, NiCo_2_O_4_ nanosheet arrays were synthesized by a modified low-temperature hydrothermal method [24-28]. Typically, 30 mmol of urea and 8 mmol of NH_4_F were dissolved completely in 30 mL deionized water, followed by the addition of 1 mmol of Ni(NO_3_)_2_ · 6H_2_O and 2 mmol of Co(NO_3_)_2_ · 6H_2_O. The mixture was transferred to a capped bottle with a FTO growth substrate facing down in the precursor at 85°C for 6 h. Once the reaction was finished, the samples were rinsed with deionized water and treated in air at 350°C for 3 h, and the NiCo_2_O_4_ nanosheet films were obtained. In the second step, to synthesize NiCo_2_S_4_ nanosheet films, the NiCo_2_O_4_ nanosheet films were put into Na_2_S · 9H_2_O solution (2 mol/L) and reacted in an autoclave at 160°C for 10 h. After the reaction, the samples were rinsed thoroughly with deionized water and dried at 60°C for 5 h in a vacuum oven.

### Fabrication of p-DSSC devices

The photocathode was prepared by immersing a NiCo_2_O_4_ sample in an ethanol solution containing 0.5 mM of N719 dye (cis-bis(isothiocyanato)bis(2,2′-bipyridyl-4,4′-dicarboxylic acid)ruthenium(II)) (Solaronix SA, Aubonne, Switzerland) for 3 h, followed by rinsing in ethanol to remove dye absorbed physically, and drying in air. The Pt counter electrode was prepared by spin coating 1 mM of chloroplatinic acid (H_2_PtCl_6_ · 6H_2_O, Aldrich, 99.9%) in 2-propanol (Sigma-Aldrich, St. Louis, MO, USA; 99.7%) onto a FTO substrate and then heating at 350°C for 30 min. The as-prepared NiCo_2_S_4_ nanosheet films were used directly as counter electrodes. The dye-coated photocathodes were sealed against Pt or NiCo_2_S_4_ counter electrodes with hot melt plastic spacers (Solaronix, 60-μm thick). The electrolyte (0.1 M LiI, 0.03 M I_2_, 0.5 M tetrabutylammonium iodide, and 0.5 M 4-tert-butylpyridine in acetonitrile) was introduced into the gap between two electrodes by a syringe. The active area of DSSCs was 0.2 cm^2^.

### Materials characterization

The morphology was characterized by field emission scanning electron microscope (FESEM; Hitachi SU8010, Hitachi Ltd., Tokyo, Japan). The microstructure was analyzed by high-resolution transmission electron microscopy (HRTEM) with selected area electron diffraction (SAED) (FEI Tecnai G2 F20 S-TWIN TMP, FEI, Hillsboro, OR, USA). The phase of products was checked by an X-ray diffractometer (XRD).

### Device measurements

Photocurrent-voltage (*J*-*V*) characteristics were performed using a Keithley 2400 SourceMeter (Keithley Instruments Inc., Cleveland, OH, USA) under simulated AM 1.5G illumination (100 mW/cm^2^) provided by a solar light simulator (94043A, Newport Corp., Irvine, CA, USA). Cyclic voltammetry (CV) and the electrochemical impedance spectroscopy (EIS) were measured with an Autolab electrochemical workstation (PGSTAT 302 N, Metrohm AG, Utrecht, The Netherlands). CV was carried out in a three-electrode system with different counter electrodes as working electrodes, a Pt foil as counter electrode, and a Ag/Ag^+^ electrode as reference electrode at a scan rate of 50 mV/s. The electrodes were immersed into an anhydrous acetonitrile solution containing 0.1 M LiClO_4_, 10 mM LiI, and 1 mM I_2_. EIS was actualized with a symmetric cell assembled with two identical counter electrodes at open-circuit voltage (*V*_oc_) bias under dark condition. The measured frequency ranged from 10 mHz to 1 MHz and the magnitude of the alternative signal was 10 mV.

## Results and discussion

**Figure 1:**
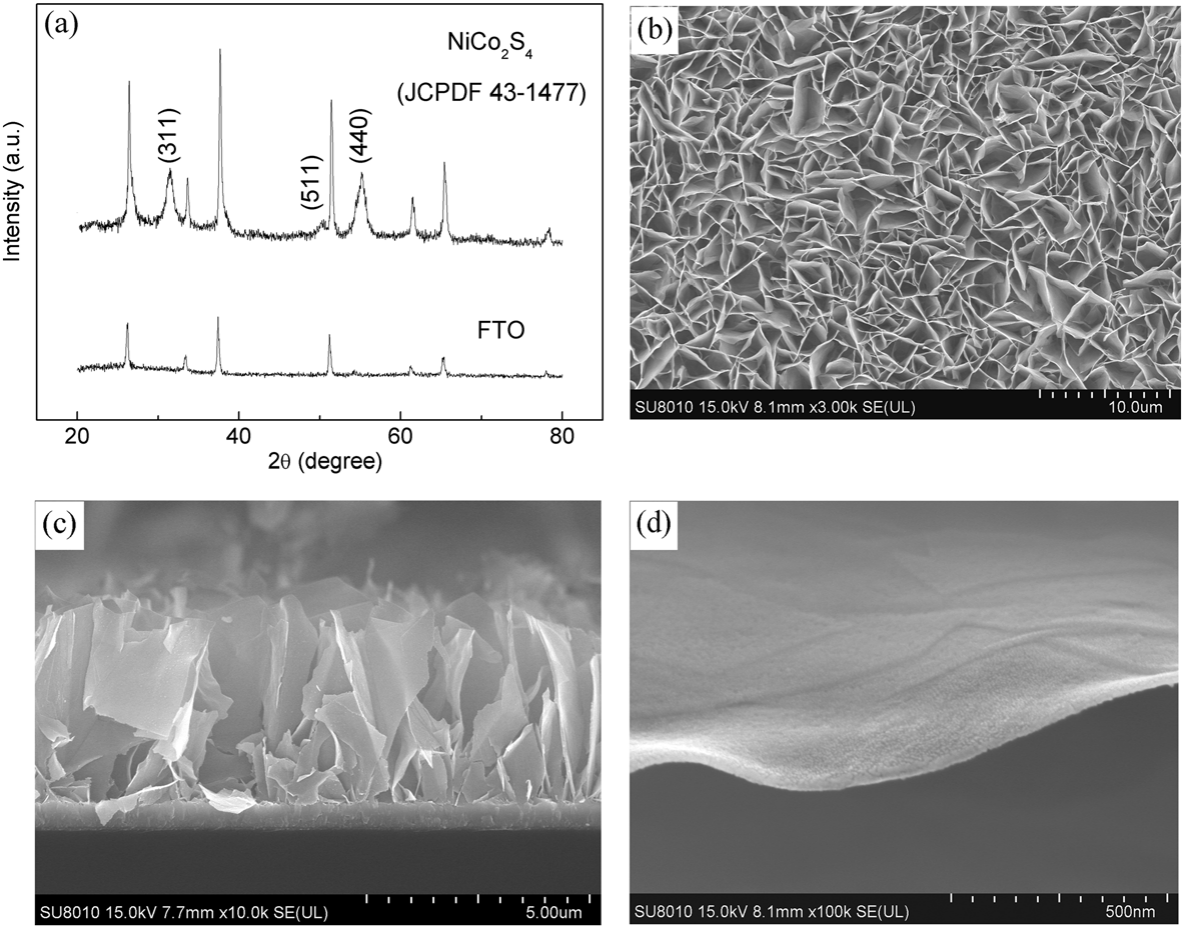
**XRD pattern and SEM images of a sample. (a)** XRD pattern of NiCo_2_S_4_ nanosheet films and representative SEM images of **(b)** large-scale and uniform growth, **(c)** cross section, and **(d)** sheet-like and porous character.

Figure 1a shows the XRD pattern of the samples after conversion process. All of the diffraction peaks can be indexed to NiCo_2_S_4_ (JCPDF card no. 43–1477). Before the ion exchange process, the diffraction peaks come from NiCo_2_O_4_ (Additional file 1: Figure S1). Figure 1b,c,d depicts typical scanning electron microscopy (SEM) images of the NiCo_2_S_4_ nanosheets. A low-magnification SEM image in Figure 1b shows a large-scale and uniform nanosheet array, and the corresponding cross-sectional image (Figure 1c) indicates that the nanosheets are vertically grown and have close contact with the FTO substrate. Higher-magnification image in Figure 1d further confirms the sheet-like character. Compared to the NiCo_2_O_4_ nanosheets (Additional file 1: Figure S2), the morphology of NiCo_2_S_4_ is almost the same and the porous surface is preserved.

**Figure 2:**
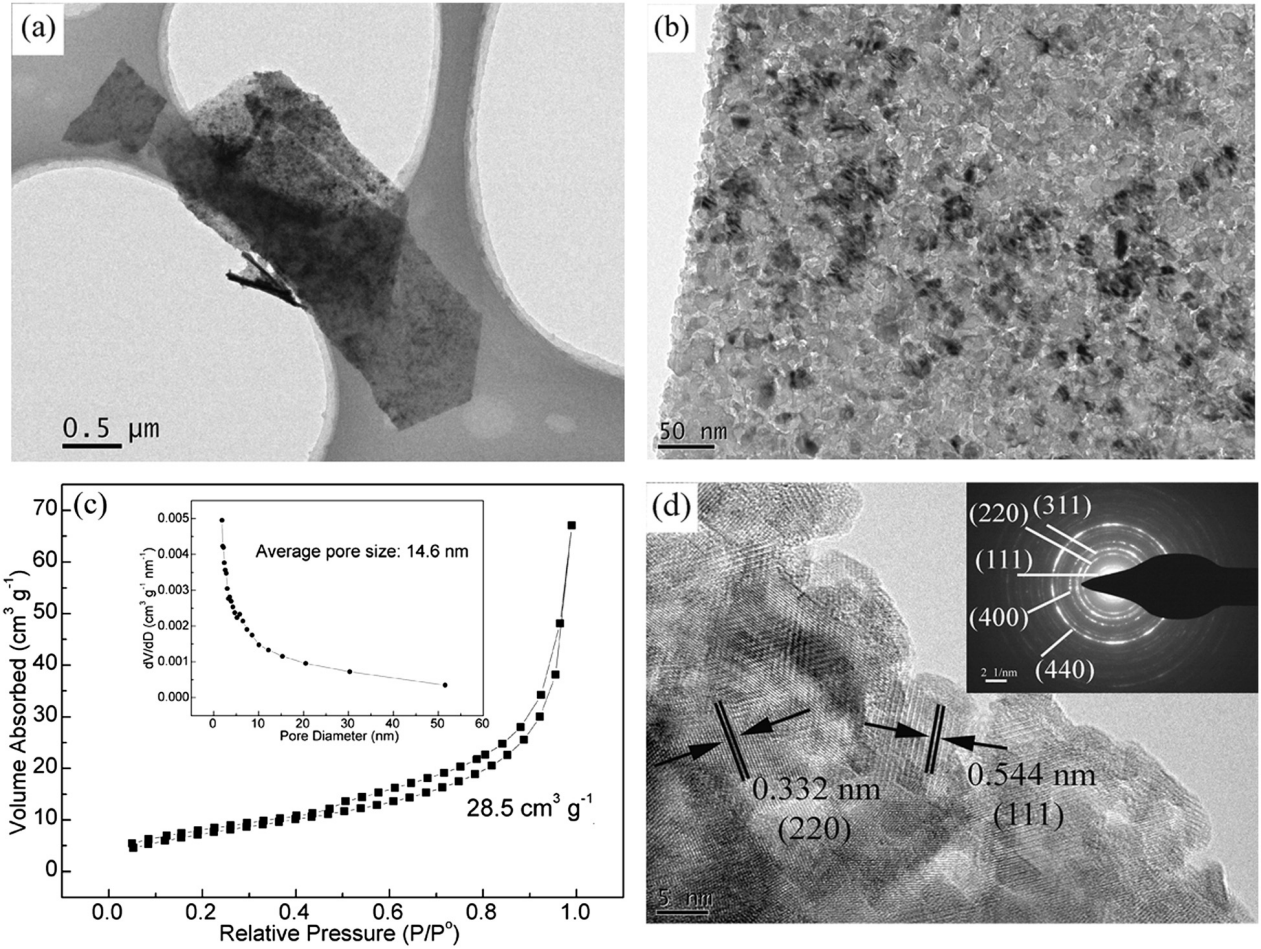
**Microstructure of the nanosheets. (a, b)** TEM images of nanosheet films, indicating that nanosheets are composed of mesopores. **(c)** N_2_ adsorption-desorption and pore-size distribution curves. **(d)** Lattice fringes and SAED pattern (inset) of the nanosheet.

The microstructure of the nanosheets is revealed by HRTEM images, as shown in Figure 2. Figure 2a,b shows that mesopores are distributed throughout the whole surface of the nanosheets. The size of the mesopores is evaluated by measuring the N_2_ adsorption-desorption curve (Figure 2c). The Brunauer-Emmett-Teller (BET) surface area of NiCo_2_S_4_ nanosheets is as high as 28.5 cm^3^/g. The pore-size distribution analysis (the inset in Figure 2c) gives an average pore size of 14.6 nm. The lattice fringes and corresponding SAED pattern (Figure 2d) indicate the polycrystalline nature of these nanosheets, and the diffraction rings can be indexed to the (111), (220), (311), (400), and (440) planes of the NiCo_2_S_4_ phase, which is consistent with the XRD result.

**Figure 3:**
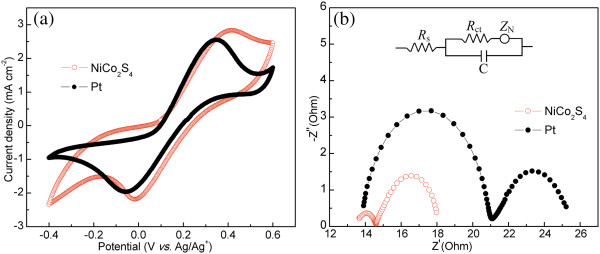
**CV curves and Nyquist plots. (a)** CV curves for the NiCo_2_S_4_ nanosheet and Pt-based counter electrodes. **(b)** Nyquist plots of EIS for symmetric cells assembled with two identical counter electrodes at an open-circuit voltage bias under dark condition.

To characterize the electrocatalytic activity of NiCo_2_S_4_ NS and Pt catalysts, CV experiments were carried out in a three-electrode system, as shown in Figure 3a. The redox reactions can be expressed by Equations 1 and 2 [29].

(1) $$3I^{-}\to \;I_{3}^{-}+\;2e^{-}\left(\mathrm{oxidation},\;\mathrm{anodic}\;\mathrm{peak}\right)$$

(2) $$I_{3}^{-}+\;2e^{-}\to \;3I^{-}\left(\mathrm{reduction},\;\mathrm{cathodic}\;\mathrm{peak}\right)$$

In the CV curves, the peak-to-peak separation (*E*_pp_) and the peak current density are two important parameters for comparing catalytic performance of different CEs [30]. The anodic current density is generally related with the rate of reaction for I^-^ oxidation in p-DSSCs. Compared to Pt CE, the NiCo_2_S_4_ NS CE possesses higher anodic and cathodic current densities, indicating that NiCo_2_S_4_ NS has higher catalytic activity than Pt. In addition, the *E*_pp_ value is inversely correlated with the standard electrochemical rate constant of a redox reaction. The *E*_pp_ of the NiCo_2_S_4_ NS CE is 0.420 V, which is almost similar with that (0.408 V) of the Pt CE. Enhanced current density and similar *E*_pp_ imply that the performance of NiCo_2_S_4_ NS CE is comparable to and even better than that of Pt CE.

EIS is a powerful tool to reveal the inherent electrochemical behaviors and the interfacial charge transfer process [31]. To further evaluate the catalytic activities of NiCo_2_S_4_ NS and Pt CEs, EIS experiments were performed using symmetric cells fabricated with two identical electrodes (CE/electrolyte/CE), as shown in Figure 3b. The Nyquist plots show two semicircles in the high-frequency (left) and low-frequency (right) regions. According to the equivalent circuit model (the inset of Figure 3b) [32,33], the high-frequency intercept on the real axis represents the ohmic series resistance (*R*_s_) outside a circuit (substrate resistance and lead connection), and the semicircle arises from the charge-transfer resistance (*R*_ct_) at the CE/electrolyte interface and the corresponding phase angle element (CPE). The low-frequency region results from the Nernst diffusion impedance (*Z*_N_) of the redox couple in the electrolyte. These parameters were obtained by fitting the Nyquist plots with the Autolab NOVA software, and corresponding results are listed in Table 1. The value of *R*_s_ for NiCo_2_S_4_ NS is almost the same with that for Pt, because the preparation procedure of two electrodes is similar. The *R*_ct_ value for NiCo_2_S_4_ NS CE is 1.25 Ω cm^2^, which is lower than that for Pt (7.32 Ω cm^2^), indicating that the former has much higher catalytic activity than the latter as evidenced by the CV tests. The value of *Z*_N_ can be calculated using Equation 3 [34]:

(3) $$Z_{N}=\;\frac{\mathrm{kT}}{n^{2}{e_{0}}^{2}\mathrm{cA}\sqrt{\mathrm{i\omega D}}}\tanh\left(\sqrt{\frac{\mathrm{i\omega}}{D}\delta }\right)\text{,}$$

**Table 1:** **Detailed photovoltaic and EIS parameters of the DSSCs with counter electrodes composed of NiCo**_**2**_**S**_**4 **_**nanosheets and Pt**

**Counter electrons**	***J***_**sc **_**(mA/cm**^**2**^**)**	***V***_**oc **_**(V)**	**FF**	***η *** **(%)**	***R***_**s **_**(Ω cm**^**2**^**)**	***R***_**ct **_**(Ω cm**^**2**^**)**	***Z***_**N **_**(Ω cm**^**2**^**)**
Pt	1.824	0.1459	59.4	0.158	13.8	7.32	4.44
NiCo_2_S_4_	2.989	0.1486	55.8	0.248	13.4	1.25	3.58

where *k* is the Boltzmann constant, *T* is the absolute temperature, *n* is the number of holes involved in the electrochemical oxidation of I^-^ at the electrode, *e*_0_ is the elementary charge, *c* is the concentration of I^-^, *A* is the electrode area, *ω* is the angular frequency, *D* is the diffusion coefficient of I^-^, and *δ* is the thickness of the diffusion layer. The *Z*_N_ values for NiCo_2_S_4_ and Pt CEs are 3.58 and 4.44 Ω cm^2^, respectively, indicating that the *D* of I^-^ in the NiCo_2_S_4_ cell is larger than that in the Pt cell. For the same electrolyte, a larger *D* means that the electrode has higher electrocatalytic activity, because faster oxidation of the I^-^ on the surface of catalysts can accelerate the diffusion of I^-^ ions in electrolyte.

**Figure 4:**
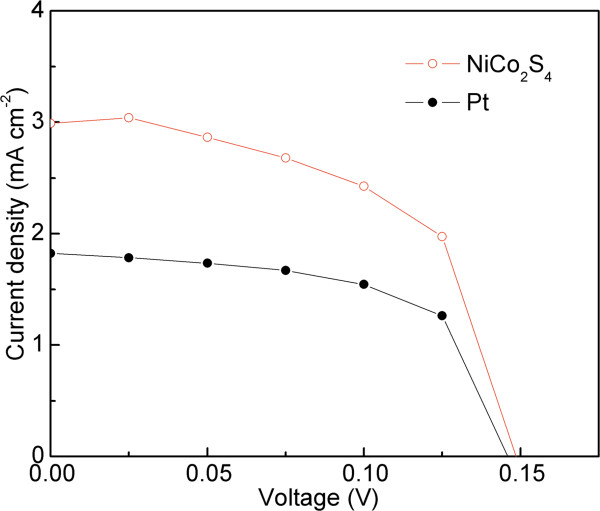
***J*-*V *curves of the DSSCs with NiCo**_**2**_**S**_**4 **_**NS and Pt CEs at 1-sun illumination.***J*-*V* characteristics of DSSCs with the NiCo_2_S_4_ nanosheet and Pt as counter electrodes, and the NiCo_2_O_4_ nanosheet as a photocathode.

Figure 4 shows the *J*-*V* curves of the DSSCs with NiCo_2_S_4_ NS and Pt CEs at 1-sun (AM 1.5G, 100 mW/cm^2^) illumination. The detailed photovoltaic parameters, including *V*_oc_, short-circuit current density (*J*_sc_), fill factor (FF), and cell efficiency (*η*) are summarized in Table 1. The DSSC with a NiCo_2_S_4_ NS CE produces an excellent cell efficiency of 0.248%, which is higher than that (0.158%) for the Pt-based reference cell. It is worthy to note that present efficiency is comparable and even higher than reported results for p-DSSCs based on typical NiO and other materials, as shown in Additional file 1: Tables S1. The *V*_oc_ value of a p-DSSC is determined by the difference between the Fermi level in the photocathode and the redox potential of I^-^/I^3-^. In the present study, there is no change in the photocathodes and the redox electrolyte; thus, the *V*_oc_ values of the DSSCs based on NiCo_2_S_4_ and Pt CEs remain almost the same. FF is mainly affected by the electron back transfer and charge recombination within devices, and series resistance, etc. Although the FF of NiCo_2_S_4_ CEs is a little lower than that of Pt CEs, the *J*_sc_ of NiCo_2_S_4_ CEs is much higher than that of Pt CEs, resulting in a higher *η*. The improved *J*_sc_ of NiCo_2_S_4_-based cell is ascribed to its mesoporous structure, which not only provides large active surface area for I^-^ oxidation but also constructs lots of open channels to facilitate the diffusion of I^-^/I^3-^ redox species. Both enhanced catalytic reaction and high diffusion rate accelerate the transfer of photogenerated holes and thus an increased current density can be expected.

## Conclusions

Low-cost NiCo_2_S_4_ NS films with high catalytic activity have been utilized as CEs of p-DSSCs based on NiCo_2_O_4_ NS photocathodes. The mesoporous nanosheets provide a large catalytically active area and facilitate the transport of I^-^/I_3_^-^ redox in electrolyte. The DSSCs with NiCo_2_S_4_ as a CE produce a higher *J*_sc_ and *η* (2.989 mA/cm^2^ and 0.248%, respectively) than those (1.824 mA/cm^2^ and 0.158%, respectively) of the cell with a Pt CE. This *J*_sc_ can almost match the performance of p-DSSCs, but the *V*_oc_ is still low. In the future, improving the *V*_oc_ by doping materials and replacing electrolyte types is an important route. The use of cost-effective NiCo_2_S_4_ as an alternative to noble Pt, in combination with a facile fabrication method, may pave the way for low-cost, scalable, and high-efficiency DSSCs.

## Competing interests

The authors declare that they have no competing interests.

## Authors’ contributions

ZS participated in the design of experiments and drafted the manuscript. HL participated in the IV analysis and revision of the manuscript. QL participated in the analysis of the CV data. FC participated in the experiment of the XRD measurements and data analysis. JG participated in the measurements and analysis of TEM. KD participated in the collection of SEM. LL participated in the design and analysis of data and revision of the manuscript. All authors read and approved the final manuscript.

## Supplementary Material

Additional file 1 **Supporting information.** This file contains XRD pattern and SEM images of NiCo_2_O_4_ nanosheets and comparison of photovoltaic parameters for the present and previously reported p-DSSCs. Click here for file
