# Supplementary material for: Efficient p-type dye-sensitized solar cells with all-nano-electrodes: NiCo2S4 mesoporous nanosheet counter electrodes directly converted from NiCo2O4 photocathodes
Source: Nanoscale Res Lett. 2014 Nov 11;9(1):608. doi: 10.1186/1556-276X-9-608 (PMC4257056; doi:10.1186/1556-276X-9-608)
Supplement: Additional file 1 — Supporting information. This file contains XRD pattern and SEM images of NiCo2O4 nanosheets and comparison of photovoltaic parameters for the present and previously reported p-DSSCs. [file 1556-276X-9-608-S1.docx]

***Supporting Information:***

**Efficient *p*-type Dye-Sensitized Solar Cells with All-Nano-Electrodes: NiCo_2_S_4_ Mesoporous Nanosheet Counter Electrodes Directly Converted From NiCo_2_O_4_ Photocathodes**

Zhiwei Shi,^†^ Hao Lu,^†^ Qiong Liu,^†^ Fengren Cao,^†^ Jun Guo,^‡^ Kaimo Deng,^†^ and Liang Li^*†^

^†^College of Physics, Optoelectronics and Energy & Collaborative Innovation Center of Suzhou Nano Science and Technology, Soochow University, Suzhou 215006, P. R. China

Email: lli@suda.edu.cn, [liang.li0216@gmail.com](mailto:liang.li0216@gmail.com)

^‡^Analysis and Testing Center, Soochow University, Suzhou, P. R. China

**
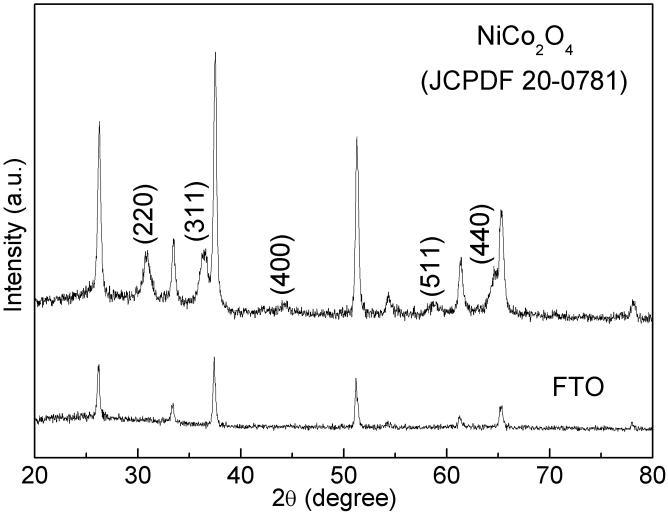
**

**Additional file 1: Figure S1**. XRD pattern of NiCo_2_O_4_ nanosheet films








**Additional file 1: Figure S2**. SEM images of NiCo_2_O_4_ nanosheets: a) large-scale growth and b) sheet-like and porous character.

| DSSCs | *J*_sc_ [mA cm^-2^] | *V*_oc_ [V] | *FF* | *η* [%] | Ref |
| --- | --- | --- | --- | --- | --- |
| NiO  CuGaO_2_ | 1.7 | 0.35 | - | 0.20 | 1 |
|  | 1.74 | 0.09 | 38 | 0.060 | 2 |
|  | 1.89-5.48 | 0.071-0.106 | 34-39 | 0.05-0.15 | 3 |
|  | 2.05 | 0.199 | 44.5 | 0.182 | 4 |
|  | 0.29 | 0.187 | 41 | 0.023 | 5 |
| CuCrO_2_ | 1.43 | 0.309 | 38 | 0.17 | 6 |
| NiCo_2_O_4_ | 2.98 | 0.148 | 55.8 | 0.248 | This work |

**Additional file 1: Table S1** Comparison of photovoltaic parameters for the present and previously reported *p*-DSSCs.

**References in Table S1.**

1 E.A. Gibson, A.L. Smeigh, L.L. Pleux, J. Fortage, G. Boschloo, E. Blart, Y. Pellegrin, F. Odobel, A. Hagfeldt, L. Hammarström, Angew. Chem. Int. Ed., **48**, 4402 (2009)

2 Z. Ji, G. Natu, Z. Huang, Y. Wu, Energy Environ. Sci., **4**, 2818 (2011)

3 L. Li, E. A. Gibson, P. Qin, G. Boschloo, M. Gorlov, A. Hagfeldt, L. Sun, Adv. Mater., **22**, 1759 (2010)

4 Z. Xu, D. Xiong, H. Wang, W. Zhang, X. Zeng, L. Ming, W. Chen, X. Xu, J. Cui, M. Wang, S. Powar, U. Bachband, Y. B. Cheng, J. Mater. Chem. A, **2**, 2968 (2014)

5 A. Renaud, B. Chavillon, L.L. Pleux, Y. Pellegrin, E. Blart, M. Boujtita, T. Pauporté, L. Cario, S. Jobic, F. Odobel, J. Mater. Chem., **22**, 14353 (2012)

6 X. Xu, J. Cui, J. Han, J. Zhang, Y. Zhang, L. Luan, G. Alemu, Z. Wang, Y. Shen, D. Xiong, W. Chen, Z. Wei, S. Yang, B. Hu, Y. Cheng, M. Wang, Sci. Rep., **4**, 3961 (2014)
